# Tenascin C-Guided Nanosystem for Precision Delivery of Obeticholic Acid in Liver Fibrosis Therapy

**DOI:** 10.3390/pharmaceutics17010032

**Published:** 2024-12-28

**Authors:** Yawen Wang, Lei Yang, Qing Xu, Taiyu Liu, Hongliang He, Lisha Liu, Lifang Yin

**Affiliations:** 1 Department of Pharmaceutics, China Pharmaceutical University, Nanjing 210009, China; 2 State Key Laboratory of Digital Medical Engineering, Jiangsu Key Laboratory for Biomaterials and Devices, School of Biological Sciences & Medical Engineering, Southeast University, Nanjing 210009, China; 3 NMPA Key Laboratory for Research and Evaluation of Pharmaceutical Preparations and Excipients, China Pharmaceutical University, Nanjing 210009, China

**Keywords:** antifibrotic nanomedicine, hepatic inflammation, TnC-triggered sequential delivery, obeticholic acid

## Abstract

**Objective:** Liver fibrosis, a hallmark of chronic liver diseases, is characterized by excessive extracellular matrix (ECM) deposition and scar tissue formation. Current antifibrotic nanomedicines face significant limitations, including poor penetration into fibrotic tissue, rapid clearance, and suboptimal therapeutic efficacy. The dense fibrotic ECM acts as a major physiological barrier, necessitating the development of a targeted delivery strategy to achieve effective therapeutic outcomes. **Methods:** We designed a liposomal delivery system functionalized with the GBI-10 aptamer and encapsulating obeticholic acid (OCA lips@Apt) to enhance selective delivery to fibrotic liver tissue while minimizing systemic toxicity. **Results:** Both in vitro and in vivo studies demonstrated that the aptamer-modified OCA liposomes effectively treated hepatic fibrosis through dual mechanisms: modulation of abnormal bile acid metabolism and attenuation of inflammation. The targeted delivery system leveraged the overexpression of Tenascin-C (TnC), a key ECM component in fibrotic tissues, for precise localization and enhanced endocytosis via the exposed cationic liposome surface. **Conclusions:** The OCA lips@Apt nanodrug demonstrated superior therapeutic efficacy with minimal off-target effects, offering a promising strategy to overcome critical barriers in liver fibrosis treatment. By precisely targeting the fibrotic ECM and modulating key pathological pathways, this TnC-guided liposomal delivery system provides a significant advancement in antifibrotic nanomedicine.

## 1. Introduction

Liver fibrosis is a severe pathological condition resulting from chronic liver injuries, characterized by the excessive accumulation of extracellular matrix (ECM) within the liver [1,2]. This debilitating state arises from various etiological factors, such as viral infections, metabolic disorders, bile acid accumulation, and non-alcoholic steatohepatitis (NASH). The underlying mechanism involves the activation of hepatic stellate cells (HSCs) into matrix-producing myofibroblast-like cells, a process driven primarily by cytokines like transforming growth factor-β (TGF-β) and platelet-derived growth factor (PDGF) [3,4,5]. Mechanistically, this activation process is critically fueled by hepatic inflammation, which serves as a key driver and a primary target for therapeutic intervention. The persistent inflammatory response in the liver not only rebuilds and reforms the ECM but also exacerbates metabolic reprogramming [6]. Conventional treatments for liver fibrosis face several limitations, including suboptimal drug delivery to fibrotic tissue and systemic side effects. To overcome these challenges, intelligent drug delivery systems with precise control over drug release have garnered significant attention. These systems offer the potential to enhance drug accumulation at the target site while minimizing off-target effects, thus improving therapeutic outcomes.

Obeticholic acid (OCA), a potent farnesoid X receptor (FXR) agonist, impacts bile acid metabolism by reducing toxic bile acid levels and enhancing their excretion, thereby alleviating inflammation and fibrosis. OCA has demonstrated significant efficacy in mitigating liver inflammation and preserving hepatocyte function. Both preclinical and clinical studies have shown that OCA treatment effectively inhibited HSC activation and prevented liver fibrosis [7,8,9]. However, the use of therapeutic OCA doses (10, 25 mg/kg daily) has been associated with dose-related adverse reactions, including pruritus, abdominal pain, and discomfort, due to the widespread activation of FXR in the gastrointestinal tract [10,11]. Furthermore, OCA’s hydrophobic nature necessitates a sophisticated delivery system to ensure proper administration.

Developing new strategies that specifically target the liver fibrosis microenvironment and enhance drug penetration is crucial to overcoming systemic toxicity and achieving effective drug delivery. Restoration of ECM homeostasis has emerged as a promising therapeutic strategy as it can counteract HSC overactivation and improve the intra-hepatic microenvironment. A key focus of our approach is the unique overexpression of Tenascin-C (TnC), a hexameric ECM glycoprotein, in fibrotic tissue, making it an ideal target for drug delivery [12]. To exploit this opportunity, the GBI-10 aptamer, with a high affinity for the TnC protein, was selected using the SELEX (Systematic Evolution of Ligands by Exponential Enrichment) method. The GBI-10 aptamer exhibits the ability to readily adsorb onto polymer materials or cationic nanoparticles through electrostatic interactions. Leveraging the GBI-10 aptamer for targeted drug delivery in fibrotic livers holds great promise in overcoming the ECM barrier and achieving cell-type preferred delivery of antifibrotic agents. This innovative approach has the potential to enhance therapeutic outcomes in liver fibrosis by improving intrahepatic drug distribution. By precisely targeting the ECM, the GBI-10 aptamer enables efficient penetration of the drug to the desired site of action [12,13]. This property facilitates the targeted delivery of therapeutic agents to fibrotic tissue, potentially overcoming the ECM barrier and achieving cell-type preferred delivery of antifibrotic agents.

Liposomes, with their excellent biocompatibility, dual hydrophilic–hydrophobic properties, and ability to encapsulate and release drugs in a controlled manner, have emerged as highly effective drug carriers. In this study, we designed ECM-responsive liposomes encapsulating OCA (OCA lips), further functionalized with the GBI-10 aptamer to form the OCA-lips@Apt nanodrug. The OCA-lips@Apt system exhibits charge-reversal properties, enabling it to respond to the highly expressed TnC in fibrotic regions. This unique design allows for enhanced accumulation, improved tissue penetration, and site-specific drug release. By specifically activating FXR-mediated antifibrotic and anti-inflammatory pathways, this platform offers a versatile strategy to delay fibrosis progression while mitigating systemic toxicity. In summary, our study presents a rationally designed OCA-lips@Apt nanodrug as a promising therapeutic platform for liver fibrosis. By addressing key challenges in drug delivery and targeting the fibrotic microenvironment, this approach has the potential to significantly improve antifibrotic therapy and advance the field of precision medicine.

## 2. Materials and Methods

### 2.1. Materials

Obeticholic acid (>98% purity, CAS: 459789-99-2) was bought from Bidepharm (Shanghai, China). Boc-Nw-(2,2,4,6,7-pentamethyldihydrobenzofuran-5-sulfonyl)-D-arginine (Boc-Arg(Pbf)) (CAS: 186698-61-3), 2-(1H-benzotriazole-1-yl)-1,1,3,3-tetramethyluronium hexafluorophosphate (HBTU) (>99% purity, CAS: 94790-37-1), 1-hydroxy benzotriazole (HOBT) (>98% purity, CAS: 200124-22-7), coumarin 6 (C6) (≥98% purity, CAS: 38215-36-0), 1,1′-dioctadecyl-3,3,3′,3′-tetramethylindocarbocyanine perchlorate (Dil) (≥98%, CAS: 41085-99-8), 1,1-dioctadecyl-3,3,3,3-tetramethylindotricarbocyanine iodide (DiR) (≥95%, CAS: 100068-60-8), MTT (methylthiazolyldiphenyl-tetrazolium bromide) (≥95%, CAS: 298-93-1), and formic acid (HPLC grade, CAS: 64-18-6) were purchased from ALLADDIN (Shanghai, China). 1,2-Distearoyl-sn-glycero-3-phosphoethanolamine (DSPE) (reagent grade, CAS: 1069-79-0), dioleoyl-sn-glycero-3-phosphocholine (DOPC) (reagent grade, CAS: 4235-95-4), and 1,2-distearoyl-sn-glycero-3-phosphoethanolamine-N-[methoxy(polyethyleneglycol)-2000] (DSPE-mPEG2000) (reagent grade, CAS: 7867-65-0) were purchased from A.V.T (Shanghai, China). N, N-diisopropylethylamine (DIPEA) (99% purity, CAS: 7087-68-5), glycyrrhetinic acid (≥98% purity, CAS: 471-53-4), lipopolysaccharide (LPS) from *E. coli* 055: B5, tetrachloromethane (CCl4) (CAS: 56-23-5), and olive oil (reagent grade, CAS: 8001-25-0) were purchased from MACKLIN (Shanghai, China). Ready-to-use DAPI staining solution was purchased from KeyGEN (Nanjing, China). The 4% Paraformaldehyde Fix Solution was purchased from Beyotime (Shanghai, China). GBI-10 aptamer and scramble GBI-10 aptamer were ordered from General Biol (Chuzhou, China). Aptamer sequences are listed in Supplementary Table S1. Specific information on Anti-Tenascin C Rabbit mAb and the β-actin antibody is detailed in Table S2.

Recombinant mouse transforming growth factor beta 1 (TGF-β1) protein was purchased from Novoprotein (Suzhou, China). Alanine aminotransferase (ALT) assay kit (C009-2-1), aspartate aminotransferase (AST) assay kit (C010-2-1), and hemoglobin test solution (C021-1-1) were acquired from Jiancheng Bioengineering Institute (Nanjing, China). Mouse IL-6 ELISA Kit and Mouse IL-1β ELISA Kit was purchased from Zeweil (Nanjing, China).

### 2.2. Cell Lines and Culture Conditions

The hepatocyte cell line AML-12 and the NIH 3T3 cells were purchased from KeyGEN Bio TECH Co., Ltd., Nanjing, China. AML-12 cells were cultured in Dulbecco’s modified eagle medium (DMEM) supplemented with 10% (*v*/*v*) fetal bovine serum (FBS) at 37 °C under 5% CO_2_. RAW264.7 macrophages and human hepatocellular carcinoma cells HepG2 cells were purchased from Procell Life Science & Technology Co., Ltd., Wuhan, China. RAW264.7 cells and NIH-3T3 cells were cultured in DMEM supplemented with 10% (*v*/*v*) FBS, 100 μg/mL streptomycin, and 100 U/mL penicillin at 37 °C under 5% CO_2_. HepG2 cells were cultured in minimum essential medium (MEM) supplemented with 10% (*v*/*v*) fetal bovine serum (FBS) at 37 °C under 5% CO_2_.

### 2.3. Animal Study

During the 4-week induction of CCl_4_, the fibrotic mice were treated with free OCA (intragastric, i.g.), OCA lips (intravenous injection), or OCA lips@Apt (intravenous injection) three times per week for two weeks (equivalent to 10 mg/kg of OCA). Mice in the healthy group and the model group were injected with PBS. At the study termination, mice were euthanized with carbon dioxide and sacrificed in accordance with the ethics committee of China Pharmaceutical University. Blood samples and major organs were collected for further analysis. The ALT and AST were determined according to the manufacturer’s instructions. Tissue samples were fixed in 10% neutral buffered formalin and processed into deparaffinized sections for hematoxylin–eosin (H&E), Sirius red, Masson’s trichrome, and TUNEL staining for pathological analysis. The remaining tissues were stored at −80 °C and homogenized for RNA and protein extraction.

### 2.4. Synthesis and Characterization of Arg-DSPE

To synthesize Arg-DSPE, 0.1 mmol of Boc-Arg (Pbf)-OH was dissolved in DMF, and DIPEA (35 μL) was added to the solution. The mixture was stirred in an ice bath for 30 min. Subsequently, 0.1 mmol of HOBT and 0.1 mmol HBTU were added while stirring. Then, 0.12 mmol of DSPE dissolved in chloroform was added dropwise to the solution at 40 °C in a water bath and allowed to react for 48 h. The reaction mixture was washed three times with brine solution and dried with anhydrous sodium sulfate. The solid crude product was purified by silica gel column chromatography using ethyl acetate:methanol:ammonia = 30:5:1 to obtain Boc-Arg-DSPE (yield: 80%). The Boc-Arg-DSPE was then dried under vacuum and dissolved in dichloromethane (DCM)/trifluoroacetic acid (TFA) for 4 h to remove tert-butyl groups. The mixture was treated with anhydrous diethyl ether, precipitating the Arg-DSPE from the reaction mixture (yield: 90%). The structure of the product was characterized using a 1H NMR spectrometer (Bruker, AVANCE500, Karlsruhe, Germany) and a TOF Mass Spectrometer (Agilent, 6230, Santa Clara, CA, USA).

### 2.5. Preparation and Characterization of OCA Lips@Apt

Firstly, the liposomes were prepared using the thin-film hydration method. Briefly, a mixture of OCA, Arg-DSPE, DOPC, and DSPE-mPEG20 (9.7:30:67:3, molar ratio) was dissolved in chloroform: methanol (3:1, *v*/*v*) in a round-bottomed flask. The organic solvents were removed by rotary evaporation under vacuum until a dried lipid film formed. The lipid film was then hydrated in pH 5.5 phosphate-buffered saline (PBS) at 55 °C for 60 min. Liposomal vesicles were obtained after sonication by using a probe sonicator, resulting in the formation of OCA lips [14]. The aptamer was then added to the OCA lips solution at different N/P ratios and vortexed for 1 min to produce OCA lips@Apt. Free OCA and unencapsulated Aptamer were eliminated using ultrafiltration tubes (MWCO 50 kDa) applying centrifugation three times at 3000 rpm for 20 min each time. Characterization of OCA lips@Apt was conducted as follows. The morphology of OCA lips@Apt and OCA lips was observed using transmission electron microscopy (TEM) (HT7700, HITACHI, Tokyo, Japan). Samples were placed on copper grids and negatively stained with 2% phosphotungstic acid before observation. The particle sizes, polydispersity index (PDI), and zeta potential were measured using a Zetasizer Nano-S90 analyzer (Malvern, UK). OCA lips and OCA lips@Apt were prepared according to the final prescription process, placed at 4 °C, and then at 1d, 2d, 3d, and 7d, respectively. The particle size of the preparations was determined, and the change in particle size was recorded. To determine the entrapment efficiency (EE) and drug loading efficiency (DLE) of OCA in OCA lips@Apt, the unencapsulated drug was removed using an ultrafiltration method. The amount of OCA was analyzed using a Triple Quad™ 5500 LC-MS/MS (AB Sciex LLC, Framingham, MA, USA) with glycyrrhetinic acid as the internal standard. Chromatographic separation was performed on an Ultimate XB-C18 (4.6 × 330 mm, 3 μm, Welch, USA) column at 40 °C. The mobile phase consisted of 0.1% formic acid in water (A) and acetonitrile (B) at a flow rate of 0.6 mL/min. The gradient elution program was 0–3.0 min, 25% B; 3.1–6.5 min, 75% B; 6.6–8.0 min, 25% B. Mass spectrometric detection was carried out using an electrospray ionization (ESI) source in negative mode, scanning range of *m*/*z* 419.3–401.4. The parameters were curtain gas 30 psi, ion spray voltage −4000 V, temperature 550 °C, declustering potential −146 V, and collision energy −46 V. The release profile of OCA lips@Apt was determined using the dialysis method. Free OCA, OCA lips, and OCA lips@Apt containing 0.15 mg of OCA were transferred to dialysis bags (MWCO 3500 Da). The bags were immersed in 30 mL PBS (pH 7.4) containing Tween 80 (10 mM) and placed in an air bath thermostat (100 rpm) for 48 h at 37 °C. Samples were collected at predetermined time points and analyzed using LC-MS/MS. The encapsulation efficiency (EE%) and drug loading efficiency (DLE %) of OCA were calculated using the following equations, respectively:

$$\mathrm{EE}\%=\frac{Mass\;of\;encapsulated\;OCA}{The\;total\;mass\;of\;OCA\;used}\times 100$$

$$\mathrm{DLE}\%=\frac{Mass\;of\;encapsulated\;OCA}{The\;total\;mass\;of\;liposomes}\times 100$$

To investigate the hemolysis effect, the blood sample was first centrifuged at 3000 rpm for 5 min, and the hematocrit was diluted with saline. The washing steps were repeated thrice until the supernatant became colorless. Saline was added into erythrocyte pellets to obtain a standard 2% erythrocyte dispersion. For the positive control, a 30 mg/mL PEI solution was incubated with 2% erythrocyte dispersion, while saline was used as the negative control. Liposomes (OCA lips or OCA lips@Apt) were incubated with 2% erythrocyte suspensions at 30 mg/mL of total lipids for 1 h. Samples were centrifuged at 2500 rpm for 5 min. The supernatant was mixed with an Hb dilution application solution and incubated in a 96-well plate for 5 min, and the absorbance was measured at 540 nm. The percentage of hemolysis was calculated for each group.

### 2.6. MTT Assay

AML-12 cells and RAW264.7 cells were seeded in 96-well plates at a density of 4 × 10^3^ cells/well in 200 μL of growth medium and incubated at 37 °C with 5% CO_2_ for 24 h. The medium was then replaced with a complete medium containing different concentrations (equivalent to OCA dose) of free OCA, OCA lips, and OCA lips@Apt, respectively. After 48 h of incubation, the cells were washed three times with PBS and 20 μL of MTT (5 mg/mL in PBS) were added to each well. The plates were incubated for an additional 4 h. Subsequently, the MTT solution was carefully removed and the formazan crystals formed by metabolically active cells were dissolved in 150 μL of DMSO. Cell viability was measured by reading the wavelength at 490 nm using a microplate reader (Shanpu Co., Ltd., Shanghai, China).

### 2.7. Internalization of OCA Lips@Apt in AML-12 and RAW264.7 Cells

Firstly, the liposomes were prepared using the thin-film hydration method. Briefly, a mixture of Dil, Arg-DSPE, DOPC, and DSPE-mPEG20 (9.7:30:67:3, *m*/*m*) was dissolved in chloroform: methanol (3:1, *v*/*v*) in a round-bottomed flask. The organic solvents were removed by rotary evaporation under vacuum until a dried lipid film formed. The lipid film was then hydrated in pH 5.5 phosphate-buffered saline (PBS) at 55 °C for 60 min. Liposomal vesicles were obtained after sonication by using a probe sonicator, resulting in the formation of Dil lips. The aptamer was then added to the lips solution at different N/P ratios and vortexed for 1 min to produce Dil lips@Apt.

AML-12 cells or RAW264.7 cells were seeded in 35 mm confocal culture dishes at a density of 1 × 10^5^ cells per dish and incubated overnight. To assess time-dependent internalization, AML-12 and RAW264.7 were cultured with Dil lips and Dil lips@Apt for 2 or 4 h, respectively. Cells were then washed three times with PBS and fixed with 4% paraformaldehyde for 15 min at room temperature. The nuclei were then stained with 4′,6-diamidino-2-phenylindole (DAPI) for 10 min in the dark. After discarding the staining solution, cells were washed three times with cold PBS. Finally, the cell samples were visualized using confocal laser scanning microscopy (CLSM) (LSM700, ZEISS, Oberkochen, Germany). Additionally, C6 lips and C6 lips@Apt were prepared according to the previously described method at a C6 concentration of 20 ng/mL and added to the wells at a C6 concentration of 100 ng/mL. The fluorescence intensity of C6 per well was quantitatively analyzed using flow cytometry (CytoFLEX, BECKMAN, Indianapolis, IN, USA).

### 2.8. Real-Time PCR and qPC

A 6-well culture plate with glass coverslips was used to seed the RAW264.7 cells with a density of 3 × 10^5^ cells per well for their exponential growth, in which the culture of RAW264.7 was kept overnight and cultured with 100 ng/mL LPS (Sigma, #L2630), which was used to induce anti-inflammation. After treatment for 12 h, total RNA from cells and tissues was extracted with an RNAeasy isolation kit (Beyotime, Shanghai, China). The extracted RNA was then converted to cDNA using the HiScript Q RT SuperMix for qPCR (+gDNA wiper) (Vazyme, Nanjing, China). The qPCR assay was conducted in technical triplicates using AceQ^®^ Universal SYBR qPCR Master Mix (Vazyme, Nanjing, China) on the LightCycler96 system (Roche, Switzerland). Results were normalized to the 18S reference gene, and gene expression was presented as fold-increase using the 2^−∆∆Ct^ method. Primer sequences are listed in Supplementary Table S3.

### 2.9. Gel Penetration Behavior

C6 Liposome was prepared according to the previously described method at a C6 concentration of 20 ng/mL. Confocal culture dishes were uniformly covered with 0.5 (*w*/ *w*) % hyaluronic acid gel. Ten minutes after the preparation was added dropwise to the confocal dish, CLSM images were taken at 200 μm intervals using a Z-stack scanning method.

### 2.10. In Vitro Penetration Behavior Within ECM-Rich 3D Multicellular Spheroids

ECM-rich 3D multicellular spheroids were created using the hanging-drop technique with methylcellulose in the medium. Briefly, 20 μL drops of a 0.24% methylcellulose-culture medium solution containing 2 × 10^4^ mixed cells (HepG2 cells and TGF-β1 activated NIH3T3 cells at a quantity ratio of 3:1) were pipetted onto the lid of 100 mm dishes containing 15 mL of PBS. Hanging drop cultures were incubated at 37 °C in 5% CO_2_ atmosphere for 3 days. The harvested 3D tumor spheroids were carefully transferred into a 12-well plate and pretreated with C6 lips, C6 lips@Apt, C6 lips@sApt (scramble aptamer modified C6 lips) at a C6 concentration of 100 ng/mL for 24 h. After washing with PBS and fixing in 4% paraformaldehyde, CLSM images were taken using the Z-stack at 10 μm intervals.

### 2.11. Fibrotic Mouse Model

Eight-week-old male ICR mice (22~25 g body weight) were purchased from SLAC laboratory (Shanghai, China). All mice animal protocols for this study were reviewed and approved by the Institutional Animal Care and Use Committee of China Pharmaceutical University (No. 202205005). The mice were housed in a temperature-controlled environment (23 ± 2 °C) with free access to a chow diet and pure water under a 12/12 h light/dark cycle. For all in vivo experiments, mice were randomly divided into different groups. To establish a CCl4-induced liver fibrosis model, 8-week-old male mice were intraperitoneally injected with CCl4 (10 mL/kg, 1:9 mixtures of CCl4 and olive oil) twice a week for 4 weeks.

### 2.12. In Vivo Biodistribution

Firstly, the liposomes were prepared using the thin-film hydration method. Briefly, a mixture of DiR, OCA, Arg-DSPE, DOPC, and DSPE-mPEG20 (9.7:30:67:3, *m*/*m*) was dissolved in chloroform: methanol (3:1, *v*/*v*) in a round-bottomed flask. The organic solvents were removed by rotary evaporation under vacuum until a dried lipid film formed. The lipid film was then hydrated in pH 5.5 phosphate-buffered saline (PBS) at 55 °C for 60 min. Liposomal vesicles were obtained after sonication by using a probe sonicator, resulting in the formation of DiR OCA lips. The aptamer was then added to the OCA lips solution at different N/P ratios and vortexed for 1 min to produce DiR OCA lips@Apt. The final DiR concentration was held constant at 50 μg/mL.

Similarly, DiD lips@Apt and DiD lips@sApt were prepared as follows: a mixture of DiD, Arg-DSPE, DOPC, and DSPE-mPEG20 (30:67:3, *m*/*m*) was dissolved in chloroform: methanol (3:1, *v*/*v*) in a round-bottomed flask. The organic solvents were removed by rotary evaporation under vacuum until a dried lipid film formed. The lipid film was then hydrated in pH 5.5 phosphate-buffered saline (PBS) at 55 °C for 60 min. Liposomal vesicles were obtained after sonication by using a probe sonicator, resulting in the formation of DiR OCA lips. The aptamer was then added to the OCA lips solution at different N/P ratios and vortexed for 1 min to produce DiR OCA lips@Apt. The final DiD concentration was held constant at 50 μg/mL.

For in vivo distribution studies, fibrotic mice were intravenously injected with free DiR, DiR OCA lips, or DiR OCA lips@Apt at a DiR dose of 0.5 mg/kg, with each formulation dispersed in saline. The mice were sacrificed, and their major organs were collected for ex vivo imaging to evaluate tissue distribution. Additionally, fibrotic mice were intravenously injected with DiD lips@Apt or DiD lips@sApt at a DiD dose of 0.5 mg/kg. Fluorescence imaging was conducted at various time points using the IVIS*^®^* Spectrum in vivo imaging system (PerkinElmer, Austin, TX, USA) to track the biodistribution of each formulation over time.

### 2.13. Enzyme-Linked Immunosorbent Assay (ELISA)

Serum levels of tumor necrosis factor-alpha (TNF-α), interleukin-6 (IL-6), and interleukin-1 beta (IL-1β) were measured using ELISA kits (Zeweil, Nanjing, China) according to the manufacturer’s instructions.

### 2.14. Statistical Analysis

The measurements used for statistical comparisons were performed on at least three biological replicates from separate experiments. Statistical analyses were carried out by the software GraphPad Prism 10.1.2, and *p* values were calculated using one-way ANOVA with Tukey’s post-hoc test. The specific statistical test used is contained in the figure legend. All data are presented as mean ± standard deviation (SD) as indicated in figure legends, and *p*-values are marked in the figures. Differences were considered significant at * *p* < 0.05, ** *p* < 0.01, *** *p* < 0.001, and **** *p* < 0.0001.

## 3. Results and Discussion

### 3.1. Preparation and Characterization of OCA Lips@Apt

**Figure 1:**
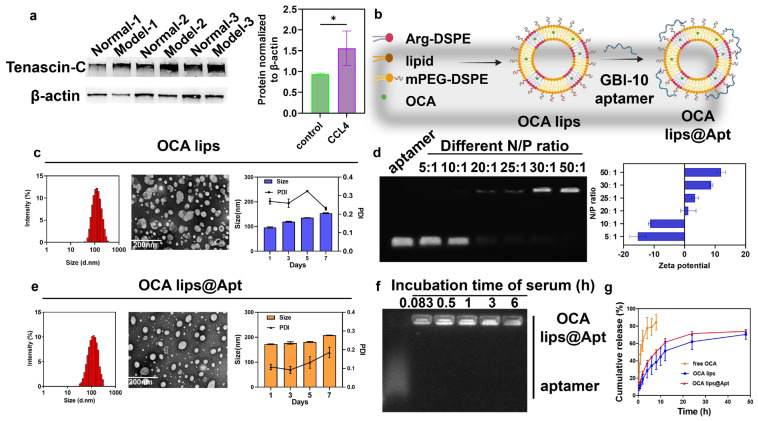
Preparation and characterization of OCA lips@Apt. (**a**) TnC expression in liver tissue by Western blot in normal and fibrotic mice. * *p* < 0.05 compared to control group. (**b**) Schematic illustration of the preparation of OCA lips and OCA lips@Apt. (**c**) Average size distribution and TEM images of OCA lips and size change of OCA lips in PBS. (**d**) Agarose gel electrophoresis of OCA lips and GBI 10 aptamer at different N:P ratios, and Zeta potential of OCA lips and GBI 10 aptamer at different N:P ratios. (**e**) Average size distribution and TEM images of OCA lips@Apt, and size change of OCA lips in PBS. (**f**) Agarose gel electrophoresis of OCA lips@Apt at different times in 10% serum. (**g**) In vitro drug release curves of free OCA, OCA lips, and OCA lips@Apt at pH 7.4 (*n* = 3).

Despite the development of various nano approaches for treating liver fibrosis, efficient drug delivery is still severely hindered by the imbalance between collagen deposition and degradation. Tenascin-C (TnC), an extracellular matrix glycoprotein, is transiently upregulated during tissue injury and plays a critical role in fibrogenesis [15]. Observations have shown that TnC protein expression is higher in fibrotic mice compared to healthy controls, confirming TnC’s role during tissue injury (Figure 1a). Thus, modifying nanomaterials with TnC-specific moieties may improve the delivery efficiency of antifibrotic agents [16,17]. In this study, we utilized negatively-charged GBI-10, the TnC-specific aptamer, to construct the lipid nanoparticle (Figure 1b).

Briefly, the cationic lipid material was designed by coupling arginine to DSPE via amid linkage, forming Arg-DSPE, which was verified by 1H-NMR spectra and mass spectrometry (Figure S1). Then, Arg-DSPE was inserted into liposomes composed of DOPC, DSPE-mPEG2000, and OCA, with the amount of Arg-DSPE optimized to achieve the appropriate particle distribution and OCA loading efficiency (Figure 1b). The resulting optimal OCA lips showed a typical liposome vesicle with a particle size of 125.93 ± 3.06 nm, a surface charge of 23.00 ± 1.31 mV, and a monodispersed spherical structure (Figure 1c). Agarose gel retardation assays revealed that the GBI-10 aptamer combined completely with the OCA lips at an N/P ratio of 1:20 (Figure 1d). OCA lips@Apt exhibited a particle size of 150.55 ± 1.36 nm, a zeta potential of 1.26 ± 2.522 mV, and a spherical shape in TEM images (Figure 1e). By calculation, OCA lips@Apt demonstrated an encapsulation efficiency (EE%) of 90.7% and a drug-loading efficiency (DLE%) of 8.25%. OCA lips@Apt exhibited good stability in PBS over 7 days, while possible coagulation or collapse occurred in OCA lips with elevated size distribution. Additionally, OCA lips@Apt maintained serum stability for 6 h without any free GBI-10 aptamer detected (Figure 1f). Furthermore, the release patterns of OCA lips and OCA lips@Apt were similar, with approximately 50% of the drug released within 8 h, reaching 70% after 24 h (Figure 1g). The release profile of OCA lips@Apt ensured the targeted release of OCA in fibrotic areas, theoretically minimizing undesired side effects in non-specific tissue.

We next characterize the in vitro biocompatibility of nanoparticles. Nanoparticle-induced hemolysis is a fundamental assay to assess the biocompatibility of designed nanocarriers [18]. The hemolysis assay revealed good compatibility of OCA lips@Apt in the blood of mice with less than a 10% relative hemolysis rate. (Figure S2). The MTT assay indicated both OCA lips and OCA lips@Apt with good biocompatibility even at a high concentration of 20 μg/mL of OCA (Figure S3).

### 3.2. Cellular Uptake and Anti-Fibrotic Performance Study In Vitro

**Figure 2:**
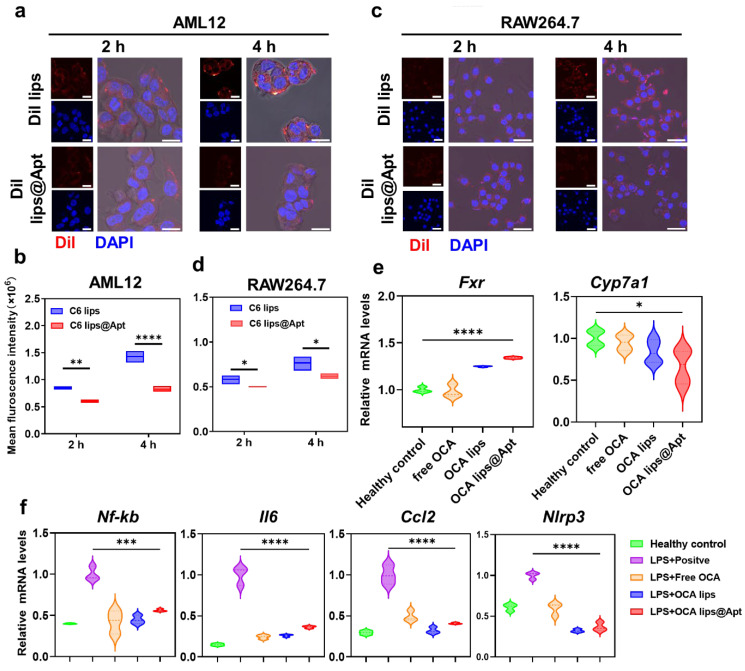
In vitro cellular uptake and antifibrotic performance of OCA lips@Apt. (**a**) Cellular uptake of Dil labeled OCA lips and OCA lips@Apt (red) into AML-12 cells after different incubation durations observed by CLSM. Scale bar = 10 µm. (**b**) Semi-quantitative analysis from flow cytometry in AML-12 cells incubated with C6 lips and C6 lips@Apt, respectively (*n* = 3). (**c**) Cellular uptake of Dil labeled OCA lips and OCA lips@Apt (red) into RAW264.7 cells after different incubation durations observed by CLSM. Scale bar = 30 µm. (**d**) Semi-quantitative analysis from flow cytometry in RAW 264.7 cells incubated with C6 lips and C6 lips@Apt, respectively (*n* = 3). (**e**) RT-qPCR analysis of *Fxr* mRNA and *Cyp7a1* mRNA in AML-12 cells after treatment with OCA-contained formulations (*n* = 3). (**f**) RT-qPCR analysis of *Nf-kb* mRNA, *Il 6* mRNA, *Ccl2* mRNA, and *Nlrp3* mRNA in LPS-pretreated RAW264.7 cells after treatment with OCA-contained formulations (*n* = 3). The statistical analysis in (**b**,**d**–**f**) was performed using a one-way ANOVA with a Turkey test. * *p* < 0.05, ** *p* < 0.01, *** *p* < 0.001, and **** *p* < 0.0001.

In the context of liver fibrosis, the accumulation of bile acids triggers hepatocyte-specific inflammatory liver injury and attracts circulating leukocytes that subsequently differentiate into monocyte-derived macrophages within the liver [19]. Our study focused on investigating the interaction between the designed OCA lips@Apt and various cell types to gain insights into hepatic uptake characteristics. To achieve this, we first incorporated Dil or C6 dyes into liposomes and examined their uptake behaviors in vitro. The CLSM images revealed the red signal surrounding the cell nucleus, indicating the internalization of both Dil lips and Dil lips@Apt into the cytoplasm of AML-12 cells (Figure 2a). Notably, the intensity of the Dil signal was higher in Dil lips compared to Dil lips@Apt, primarily due to the positive surface potential that facilitated efficient uptake by hepatocytes. Additionally, the intensity of Dil fluorescence increased over a 4 h incubation period with AML-12 cells, highlighting a time-dependent uptake pattern. These observations were further verified by flow cytometry analysis (Figure 2b). It is reported that the macrophages in the liver tend to uptake negative-charged liposomes rather than the released positively-charged OCA lips. However, in this study, we observed that the RAW 264.7 cells treated with Dil lips showed weaker fluorescence than the positive-charged Dil lips@Apt after 2 h incubation, which can be attributed to the modification of the GBI-10 aptamer. This also implied that OCA lips@Apt could decrease non-specific uptake by Kupffer cells within the liver (Figure 2c,d).

Given the uptake characteristics of nanoparticles, we next explored the potential anti-fibrotic mechanism of OCA lips@Apt to ensure therapeutic efficacy in vivo. In liver fibrosis, impaired regulation of bile acid (BA) metabolism leads to the buildup of harmful BA species within hepatocytes [20]. This accumulation triggers inflammation, oxidative stress, and hepatocellular injury, contributing to the progression of fibrosis. Disturbances in BA transporters, such as the downregulation of the bile salt export pump (BSEP), exacerbate the toxic effects of accumulated BA [20]. Therefore, maintaining BA homeostasis is crucial for effective liver fibrosis treatment. Several FXR modulators have been developed to address liver disorders associated with BA and lipid accumulation [21]. To assess the impact of OCA on hepatocytes, we examined the gene expression related to bile acid metabolism. Our results demonstrated that both OCA lips and OCA lips@Apt effectively activated the expression of FXR (Figure 2e). Encouragingly, treatment with OCA lips@Apt resulted in significant repression of the gene expression of cholesterol 7α-hydroxylase CYP7A1, indicating its potential to protect against hepatocyte damage and halt liver fibrosis progression.

Mechanistically, FXR activation has also been involved in the inflammatory response, likely through the repression of NF-κB signaling and the NLRP3 inflammasome. To elucidate the anti-inflammatory effect of FXR activation induced by different OCA treatments, we utilized LPS-primed mouse macrophages (RAW264.7). Remarkably, OCA lips@Apt treatment led to downregulation of NF-κB, IL6, CCL2, and NLRP3 levels in RAW264.7 cells preincubated with LPS, suggesting an enhanced anti-inflammatory response (Figure 2f). These findings collectively support the rationale for synergistically regulating bile acid homeostasis and the inflammatory response to amplify therapeutic outcomes.

### 3.3. Sequential Delivery of OCA Lips@Apt in Fibrotic Model

**Figure 3:**
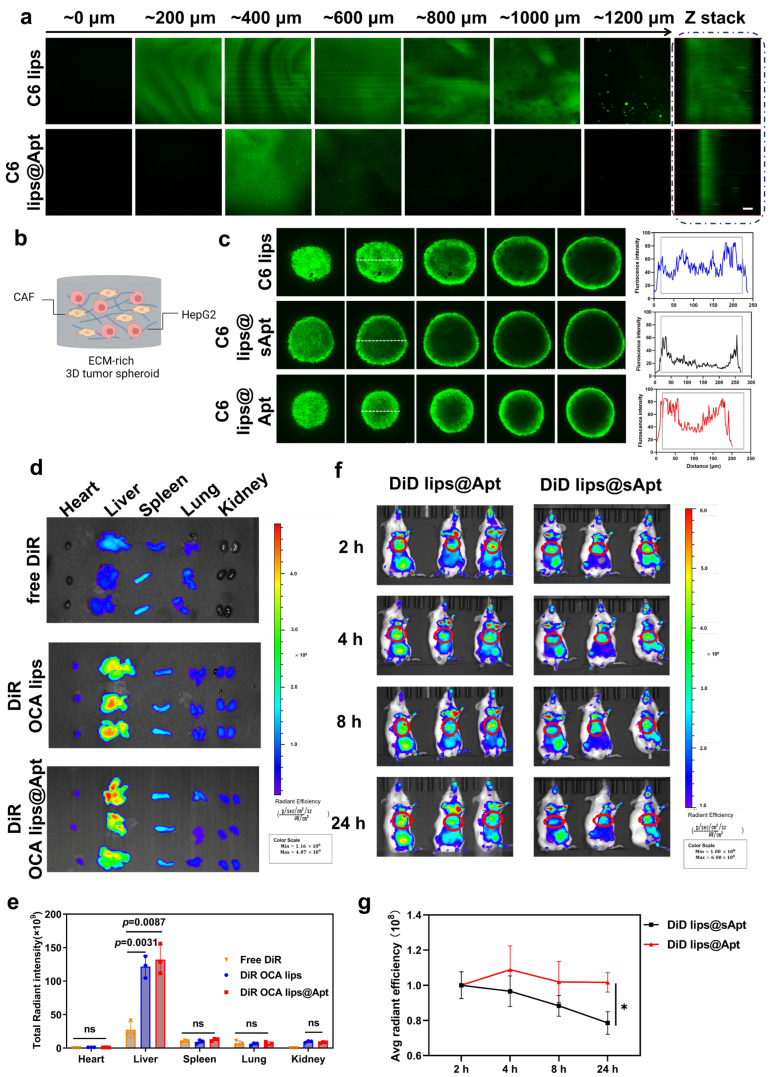
In vitro penetration study of OCA lips@Apt and tissue distribution behavior in fibrotic mice. (**a**) C6 lips and C6 lips@Apt were added to the gel layer and scanned using CLSM Z-stack. Scale bar = 100 μm (**b**) Schematic illustration of the construction of multicellular ECM-rich 3D multicellular spheroids. (**c**) In vitro penetration of C6 lips, C6 lips@sApt, and C6 lips@Apt in HepG2/TGF-β1 pre-treated NIH-3T3 cocultured tumor spheroids observed by CLSM, scale bar = 100 µm. Semi-quantification results of the image (white dash line). (**d**) A total of 24 h after fibrotic mice were intravenously injected with DiR and DiR-loaded liposomes, the fluorescence intensities of vital organs were monitored using an IVIS Spectrum instrument. (**e**) Fluorescence intensity of each organ was calculated accordingly (*n* = 3). (**f**) Representative in vivo IVIS spectrum pictures of fibrotic mice treated with different formulations. ns, not significant. (**g**) Fluorescence intensity of each mouse was calculated accordingly (red circle). * *p* = 0.0134 compared with OCA lips@Apt. The statistical significance was assessed using a one-way ANOVA with a Tukey test.

Excessive deposition of ECM constructs the pathological barrier that restricts the delivery of nanoparticles [22]. To investigate how OCA lips@Apt penetrate the ECM barrier in vitro, we conducted an experiment by using ECM-mimicking hydrogels and a 3D multicellular spheroid model. Initially, we prepared 0.5 (*w*/*w*) % hyaluronic acid-based hydrogel to replicate the dense ECM environment typical of fibrotic liver tissue. C6 lips were added to the hydrogel, and their diffusion was observed by fluorescence microscopy. Our findings indicate that the positively charged C6 lips demonstrated significant penetration ability compared to the negatively charged C6 lips@Apt (Figure 3a). Fluorescence intensity measurements demonstrated a significant increase in the penetration depth of C6 lips, emphasizing their efficient passage through the ECM (Figure 3a). This enhanced penetration may be due to the segregation of the GBI-10 aptamer, exposing the positively charged OCA lips and facilitating their interaction and uptake with target cells embedded in the ECM.

This variability in penetration may be due to the fact that positively charged C6 lips more readily penetrate the negatively charged hydrogel, whereas the lack of TNCs in the hydrogel results in the inability of the aptamer to dissociate from the C6 lips, and the agent remains negatively charged, which in turn contributes to the weaker penetration effect. To further elucidate how OCA lips@Apt interact with the ECM, we employed a 3D multicellular spheroids model composed of TGF-β1-pretreated NIH 3T3 cells and HepG2 cells (Figure 3b). Previous studies have established that TGF-β upregulates TnC gene expression and protein levels under various pathological conditions. Here, we confirmed increased TnC expression following pretreatment of NIH 3T3 cells with 10 ng/mL TGF-β1 (Figure 3b). In our experimental setup, C6 served as a fluorescent probe within OCA lips and OCA lips@Apt to create C6 lips and C6 lips@Apt, respectively, while C6 lips@sApt with the scramble GBI-10 aptamer served as a control. The 3D spheroids were incubated with these liposomal formulations, and their distribution was observed using CLSM Z-stack scanning (Figure 3c). The images revealed significantly enhanced penetration efficacy in the C6 lips group, likely attributed to the positive surface charge of Arg-DSPE. In contrast, C6 lips@Apt exhibited penetration into the interior regions of the multicellular spheroids, whereas C6 lips@sApt primarily localized in the peripheral regions of the tumor spheroids. Such a phenomenon might be due to the different bioeffects of the scrambled aptamer (sApt) and tenascin-C affinity aptamer GBI-10. The GBI-10 aptamer has a specific affinity for TnC proteins, and the aptamer separates from liposomes [23], thereby facilitating the uptake of exposed positively charged C6 lips.

Having established the TnC-triggered penetrating ability of OCA lips@Apt in vitro, our next aim was to investigate its biodistribution in the context of a fibrotic liver. The ex vivo imaging showed that the stronger fluorescence signal of both DiR liposomes and DiR lips@Apt in fibrotic livers, compared to that of the free DiR group (Figure 3d,e), which makes the nanoparticle passively accumulate in the liver. To gain insights into the ECM penetration behavior of OCA lips@Apt in vivo, we used DiD-labeled OCA lips@Apt (DiD lips@Apt) and DiD-labeled OCA lips@sApt (DiD lips@sApt). The DiD lips@Apt displayed significantly higher fluorescence in the liver 24 h post-injection, allowing the enhanced accumulation effect within the liver (Figure 3f,g). These results confirm that the GBI-10 modification circumvents the ECM barrier and improves intrahepatic delivery for fibrosis therapy.

### 3.4. Antifibrotic Activity in Liver Fibrosis Model

Anti-fibrotic therapies can generally be categorized based on their mechanisms, including (1) hepatocyte protection, (2) immune response modulation, or (3) hepatic stellate cell (HSC) inactivation [20]. Despite extensive research into liver fibrosis, there are currently no approved drugs specifically targeting this condition. Our study aimed to explore the potential of OCA lips@Apt in modulating the intricate intra-hepatic crosstalk between bile acid metabolism and the inflammatory response. By elucidating the interplay between these key processes, we hope to contribute to the development of novel therapeutic strategies for liver fibrosis treatment.

**Figure 4:**
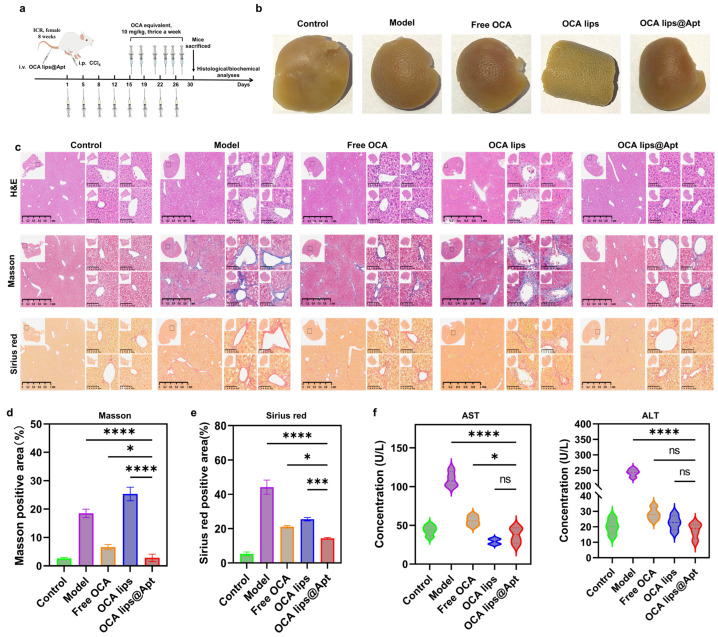
In vivo antifibrotic efficacy in CCl4-induced fibrosis model. (**a**) Schedule of the therapeutic regimen of OCA lips@Apt. (**b**) Representative photographs of normal and fibrotic livers of mice. Livers were collected at 24 h post-injection and perfused with PBS buffer as well as fixed with 4% paraformaldehyde. (**c**) Representative images of liver photos, H&E (5×, scale bar = 1mm, 20×, scale bar = 100 μm), Masson (5×, scale bar = 1mm, 20×, scale bar = 100 μm), and Sirius Red (5×, scale bar = 1mm, 20×, scale bar = 100 μm) of liver sections of mice with different treatments. (**d**,**e**) Semiquantitative analysis of the area of Masson and Sirius Red staining sections. (**f**) Liver enzyme assay of AST and ALT of mice with different treatments (*n* = 3). The statistical significance was performed using a one-way ANOVA with a Tukey test. *p* values: ns, not significant, * *p* < 0.05, *** *p* < 0.001, **** *p* < 0.0001.

In this study, fibrotic mice were subjected to a two-week treatment regime involving free OCA, OCA lips, and OCA lips@Apt (Figure 4a). At the experimental endpoint, the mice were euthanized, and their liver tissues were isolated for the evaluation of fibrosis-related biomarkers. The liver’s texture appeared coarse, nodular, and uneven after CCl4 induction due to the fibrosis progress, while the livers obtained from the OCA lips@Apt group exhibited a smoother texture (Figure 4b). Subsequently, the extent of fibrosis was assessed using histological techniques, including H&E staining, Masson’s trichrome staining, and Sirius red staining. H&E staining revealed that livers obtained from healthy mice displayed a normal lobular architecture, while those from CCl4-induced mice exhibited significant fibrous septa formation and lymphocyte infiltration. Treatment with OCA lips@Apt for two weeks resulted in improved histological parameters, with a notable reduction in micro-vesicular steatosis and infiltrating inflammatory cells (Figure 4c). Masson’s trichrome staining showed abundant collagen deposition in the livers of the fibrotic model mice, while healthy mice exhibited minimal collagen deposition, highlighting the beneficial contribution of GBI-10 aptamer modification in enhancing intrahepatic delivery. Semi-quantitative calculation of Masson positive staining area indicated a remarkable decrease in collagen area in OCA lips@Apt treated mice compared to free OCA and OCA lips treatment (Figure 4d). By contrast, OCA lips treatment induced more severe fibrosis, suggesting that it might trigger the inflammatory response. Sirius red staining further confirmed extensive scar tissue formation in fibrotic mice, which was significantly reduced after OCA lips@Apt treatment (Figure 4e). The minimal antifibrotic effects observed with OCA lips alone highlight the importance of targeted intrahepatic delivery. TUNEL assays revealed increased hepatocyte apoptosis following CCl_4_ induction, which was significantly attenuated by OCA lips@Apt treatment, demonstrating a protective effect against hepatocyte injury (Figure S4). Additionally, the biosafety assay was evaluated by histological analysis of major organs, with H&E-stained sections showing no discernible focal lesions in mice treated with free OCA, OCA lips, or OCA lips@Apt (Figure S5). The critical serological markers of liver function, such as ALT and AST, were measured to evaluate efficacy. All indicators in the fibrotic model group were elevated compared to the healthy control group. All treatments containing OCA significantly reduced the serum levels of ALT and AST (Figure 4f). Collectively, these findings underscore the potential of the GBI-10 aptamer as a promising targeting moiety to improve the intrahepatic delivery of OCA for resolving liver fibrosis. This nanosystem not only restores liver function but also exhibits favorable biocompatibility in vivo.

### 3.5. OCA Lips@Apt Attenuates Liver Fibrosis Through FXR Signaling Pathway and ECM Remodeling

**Figure 5:**
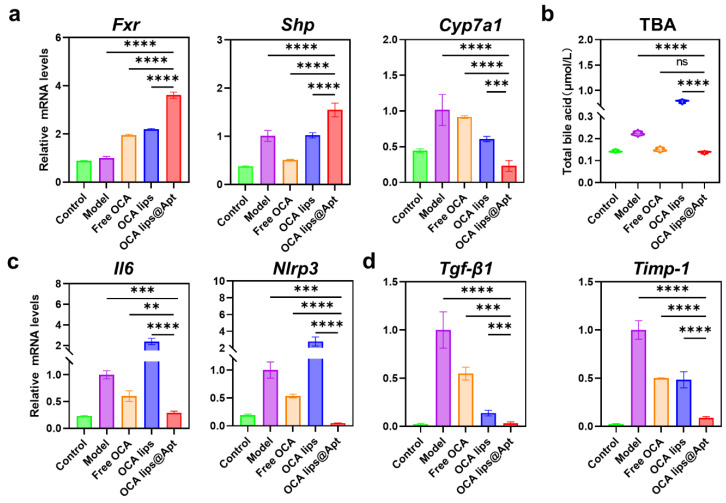
OCA lips@Apt act as potent FXR agonist in vivo. (**a**) RT-qPCR assay for monitoring *Fxr* mRNA, *Shp* mRNA, and *Cyp7al* mRNA in livers of fibrotic mice treated with various formulations (*n* = 3). (**b**) Levels of serum total bile acids in various groups (*n* = 3). (**c**) RT-qPCR assay for monitoring *Il6* and *Nlrp3* mRNA in livers of fibrotic mice treated with various formulations (*n* = 3). (**d**) RT-qPCR assay for monitoring *Tgf-β* and *Timp-1* mRNA in livers of fibrotic mice treated with various formulations (*n* = 3). The statistical significance was assessed using one-way ANOVA with the Tukey test. *p* values, ** *p* < 0.01, *** *p* < 0.001, **** *p* < 0.0001, ns, not significant.

To explore the antifibrotic mechanisms in more depth, we meticulously collected liver samples and conducted a series of bioassays. First, we comprehensively assessed the effects on the farnesol X receptor (FXR) signaling pathway and bile acid metabolic homeostasis. OCA, as a potent agonist of FXR, induces the nuclear receptor small heterodimeric chaperone (SHP), which inhibits the transcription of CYP7A1, the rate-limiting enzyme of bile acid synthesis [24]. In our study, we assessed FXR activity following different treatments (Figure 5a). Notably, OCA lips@Apt exhibited a more pronounced FXR activation compared to other treatment groups. We observed significant suppression of CYP7A1 expression in livers treated with OCA lips@Apt, indicating the GBI-10 aptamer guided delivery of the OCA to the fibrotic liver. In addition to suppressing CYP7A1, FXR activation enhances the expression of a small heterodimer partner (SHP), an atypical nuclear receptor that acts as a transcriptional repressor involved in BA synthesis. SHP directly inhibits the expression of liver receptor homolog-1 (LRH-1), a key transcriptional factor required for the hepatic CYP7A1 expression. Remarkably, the expression level of SHP was significantly diminished in the targeted intrahepatic delivery of the OCA lips@Apt compared to free OCA and OCA lips. We also measured the levels of total bile acids (TBAs). OCA Lip@Apt significantly decreased TBA levels, while OCA Lip elevated them (Figure 5b). These data reveal that OCA lips@Apt restored BA homeostasis, prevented the accumulation of toxic BA species, and consequently alleviated liver cell damage, thereby delaying the progression of liver fibrosis.

The interplay between bile acid metabolism, immune cell activation, and fibrogenesis underscores the complexity of liver fibrosis and its association with inflammatory responses [25]. As has been demonstrated, FXR activation attenuates the inflammatory response by reducing the release of proinflammatory cytokines during the early phases of fibrosis [26]. These cytokines play a crucial role in the recruitment and activation of monocyte-derived inflammatory cells, thereby exacerbating liver injury. RT-qPCR analysis revealed that intrahepatic delivery of OCA lips@Apt significantly reduced the mRNA expression of the targeted genes compared to the untreated group (Figure 5c). Recent studies have also shown that OCA selectively blocks the NLRP3 inflammasome independently of FXR activation, further contributing to the reduction in the inflammatory response. As expected, the lower expression of proinflammatory cytokines and the inhibition of inflammasome activation upon OCAlips@Apt treatment, compared to the free OCA and OCA lips groups, further support our hypothesis. In contrast, OCA lips triggered a pronounced inflammatory response, as evidenced by higher mRNA expression levels. This inflammatory response likely results from non-specific interactions between OCA lips and the immune system. Without aptamer modification, OCA lips lack targeting specificity, leading to off-target accumulation in macrophage-rich tissues (e.g., liver and spleen), activation of immune cells, and subsequent elevated release of proinflammatory cytokines.

The phenotypic transformation of quiescent H SCs is widely acknowledged as a pivotal event in liver fibrosis [27]. We detected and analyzed the gene expression of some fibrosis-related biomarkers. RT-qPCR results showed that gene expressions of TGF-β1 and Timp-1 were significantly elevated after four weeks of CCl4 induction, which is in line with the histological and immunohistochemistry staining images (Figure 5d). The excessive deposition of ECM is closely associated with the secretion of pro-fibrotic cytokines, such as TGF-β1, which plays a critical role in stimulating the transition from dormant to activated HSCs by initiating Smad3 phosphorylation. The expression of TGF-β1 was markedly reduced following treatment with OCA lips@Apt, suggesting a potential role for OCA in modulating the interaction between FXR agonism and the TGF-β1 signaling pathway. Additionally, studies have shown the balance between MMPs and TIMPs acts as a critical regulator in the ECM reconstruction and, subsequently, the progression and regression of hepatic fibrosis [28,29]. While this study has several strengths, it is important to take into consideration certain limitations inherent to the current study. Although OCA lips@Apt demonstrated a significant resolution on liver fibrosis in the early stage, it is essential to evaluate the efficacy study on advanced stages or other liver fibrosis-related animal models (e.g., NAFLD, NASH). Additionally, the liver-targeting mechanism of OCA lips@Apt needs to be further studied to determine the interaction of OCA lips@Apt and TnC protein in vivo. Because aptamer-functionalized liposomes interact with various molecules in the body, comprehensive pharmacokinetic studies are essential. These studies may provide insights into the safety, circulation, biodistribution, and clearance of aptamer-functionalized liposomes, particularly when administered via intravenous injection.

Overall, these results demonstrate that OCA lips@Apt not only modulate bile acid metabolism but also possess the ability to resolve inflammation, providing a multifaceted anti-fibrotic effect in the liver. This approach holds great promise for the treatment of liver fibrosis.

## 4. Conclusions

In summary, our study presents a novel approach for targeted drug delivery in liver fibrosis using GBI-10 aptamer-coated charge-reversal liposomes. Through the rational design of the OCA lips@Apt nanodrug, we demonstrate the potential to overcome systemic toxicity and intrahepatic delivery obstacles. This therapeutic platform holds significant promise for improving treatment outcomes in liver fibrosis by ensuring precise delivery and efficient action of antifibrotic agents within the liver. Future research will focus on further optimizing this delivery system, exploring its efficacy in preclinical and clinical settings, and investigating its potential applications in other fibrotic diseases. This approach represents a significant advancement in the field of nanomedicine and offers new hope for patients suffering from liver fibrosis.

## Data Availability

The data presented in this study are available on request from the corresponding author. The data are not publicly available due to limited web resources.

## Supplementary Materials

The following supporting information can be downloaded at: https://www.mdpi.com/article/10.3390/pharmaceutics17010032/s1, Figure S1. Synthesis route and structural characterization of Arg-DSPE. (a) Synthetic route of Arg-DSPE. (b) ^1^H-NMR spectrum of Arg-DSPE in CDCl_3_ and TOF Mass Spectrometry of Arg-DSPE in MeOH. Figure S2. Hemolysis induced negative control, positive control, OCA lips, and OCA lips@Apt on the murine erythrocytes (left) and quantification of the relative hemolysis analysis (right). * *p* < 0.0001 vs. positive control group. The statistical significance was assessed using a one-way ANOVA with Turkey test. Results are presented as mean ± SD (*n* = 3). Figure S3. Cytotoxicity of free OCA, OCA lips, and OCA lips@Apt with the concentration of OCA ranging from 0.001 to 20 μg/mL in AML-12 (a) and RAW264.7 cells (b) by MTT assay. Figure S4. Representative images of TUNEL staining liver tissues sections. Nuclei were counterstained with DAPI (blue). Scale bar = 100 μm. Figure S5. The representative images of H&E staining of vital organs (heart, spleen, lung and kidney) after various treatments. Scale bar = 100 μm. Table S1. Aptamer sequences. Table S2. Antibody information. Table S3. PCR primer sequences.
