# Supplementary material for: Tenascin C-Guided Nanosystem for Precision Delivery of Obeticholic Acid in Liver Fibrosis Therapy
Source: Pharmaceutics. 2024 Dec 28;17(1):32. doi: 10.3390/pharmaceutics17010032 (PMC11768695; doi:10.3390/pharmaceutics17010032)
Supplement: Supplementary file 1 [file pharmaceutics-17-00032-s001.zip › pharmaceutics-3382475-supplementary.pdf]

Supplementary Information

Tenascin C-Guided Nanosystem for Precision Delivery of Obeticholic Acid in Liver Fibrosis Therapy

Yawen Wang [1, †], Lei Yang [1, †], Qing Xu [1], Taiyu Liu [1], Hongliang He [3], Lisha Liu [1, 2, *], and Lifang Yin [1, 2, *]

[1] *Department of Pharmaceutics, China Pharmaceutical University, Nanjing 210009, China*

[2] *NMPA Key Laboratory for Research and Evaluation of Pharmaceutical Preparations and Excipients, China Pharmaceutical University, Nanjing 210009, China*

[3] *State Key Laboratory of Digital Medical Engineering, Jiangsu Key Laboratory for Biomaterials and De-vices, School of Biological Sciences & Medical Engineering, Southeast University, Nanjing, 210009*

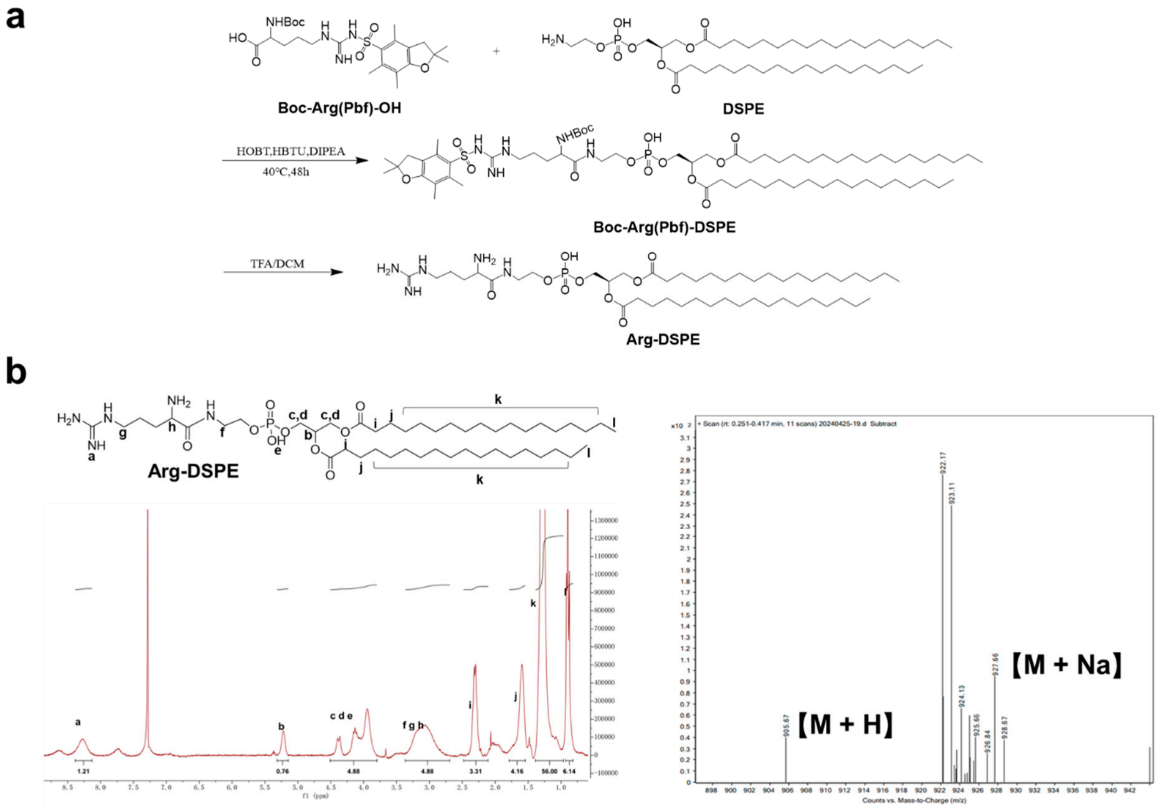

Figure S1 Synthesis route and structural characterization of Arg-DSPE. a) Synthetic route of Arg-DSPE. b) ^{1}H-NMR spectrum of Arg-DSPE in $CDCl_3$ and TOF Mass Spectrometry of Arg-DSPE in MeOH.

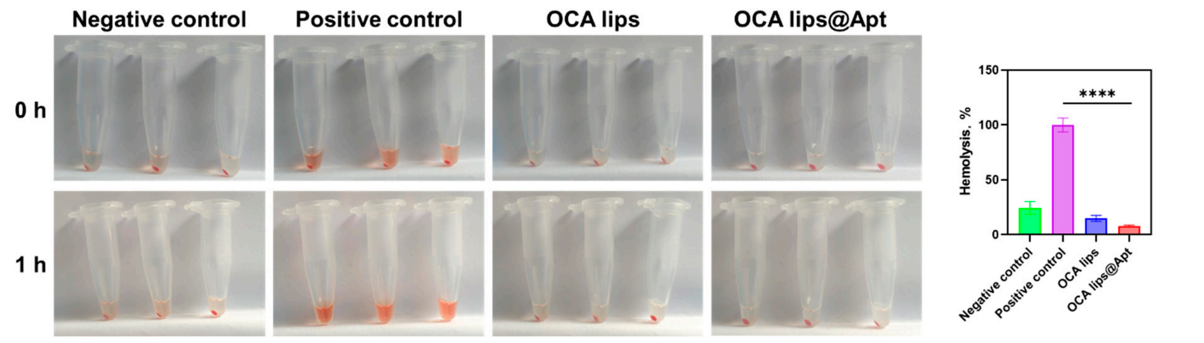

Figure S2 Hemolysis induced negative control, positive control, OCA lips, and OCA lips@Apt on the murine erythrocytes (left) and quantification of the relative hemolysis analysis (right). *p < 0.0001 vs. positive control group. The statistical significance was assessed using a one-way ANOVA with Turkey test. Results are presented as mean ± SD (n=3).

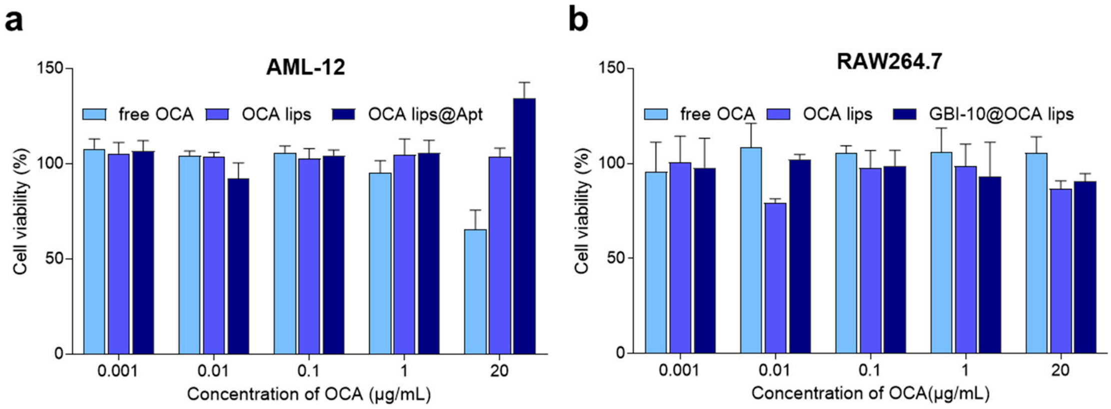

Figure S3 Cytotoxicity of free OCA, OCA lips, and OCA lips@Apt with the concentration of OCA ranging from 0.001 to 20 μg/mL in AML-12 (a) and RAW264.7 cells(b) by MTT assay.

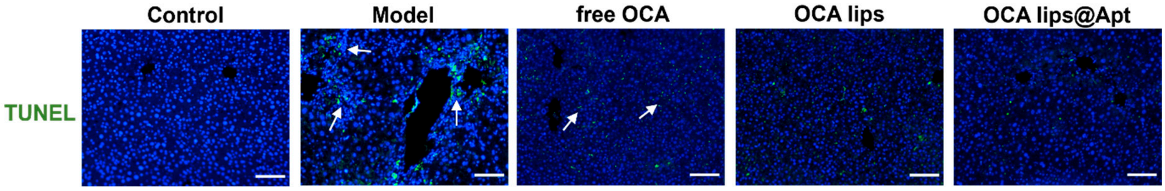

Figure S4 Representative images of TUNEL staining liver tissues sections. Nuclei were counterstained with DAPI (blue). Scale bar = 100 μm.

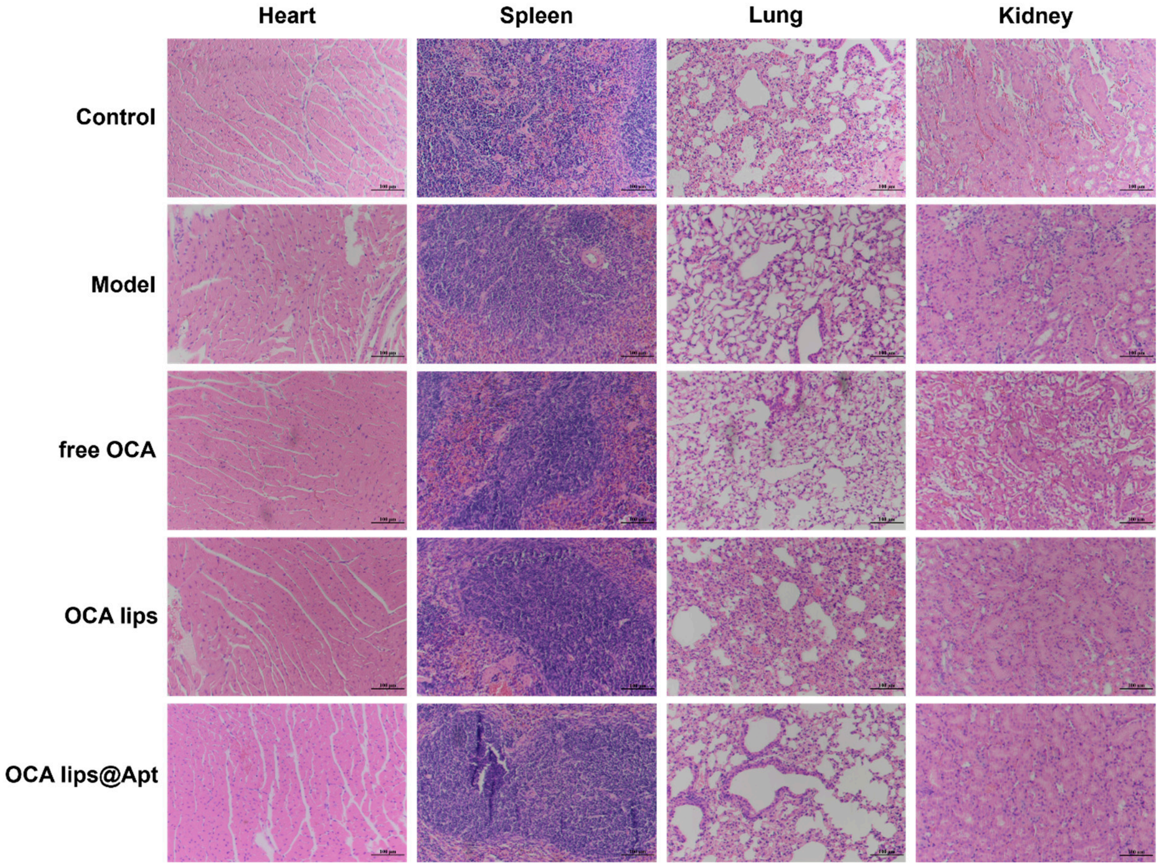

Figure S5 The representative images of H&E staining of vital organs (heart, spleen, lung and kidney) after various treatments. Scale bar = 100 μm.

Table S1 Aptamer sequences

Genes	Sequence (5’-3’)
GBI-10 aptamer	CCCAGAGGGAAGACTTTAGGTTCGGTTCACGTCC
scramble GBI-10 aptamer	ATCTTGAGCTTACGGCCAAGCAGTTTCCGCGGAG

Table S2 Antibody information

**Target**	**Host**	**Source and identifier**	**Application**
Tenascin C	Rabbit	Abcam, ab271877	WB
β-actin	Mouse	Santa Cruz, sc-47778	WB

Table S3 PCR primer sequences

Genes	Forward primer (5'-3')	Reverse primer (5'-3')
Mouse *18s*	TAGAGGGACAAGTGGCG	ACGCTGAGCCAGTCAGTGTA
Mouse *Fxr*	GGCAGAATCTGGATTTGGAATCG	GCCCAGGTTGGAATAGTAAGACG
Mouse *Shp*	CGATCCTCTTCAACCCAGATG	AGGGCTCCAAGACTTCACACA
Mouse *Cyp7a1*	TACTAGATAGCATCATCAAGGAGGCTC	CCATCCTCAAGGTGCAGAGTG
Mouse *Bsep*	TTCTGTTCTCCACCACTA	GCCATAATGAAGTTGTATGC
Mouse *Nf-κb*	AGGCTTCTGGGCCTTATGTG	TGCTTCTCTCGCCAGGAATAC
Mouse *Il6*	TAGTCCTTCCTACCCCAATTTCC	TTGGTCCTTAGCCACTCCTTC
Mouse *Ccl2*	CAGGTCCCTGTCATGCTTCT	GTCAGCACAGACCTCTCTCT
Mouse *Nlrp3*	AAAGAGGAAGTCAACCCTTGGACC	CACCAGTCTGGAAGAACAGGCAAC
Mouse *Timp-1*	CCAGAACCGCAGTGAAGAGTT	AGAGTACGCCAGGGAACCA
Mouse *Tgf-β*	TGGAGCAACATGTGGAACTC	TGCCGTACAACTCCAGTGAC
Mouse *α-sma*	GGCACCACTGAACCCTAAG	AATACCAGTTGTACGTCCAGA
